# Serum 25-hydroxyvitamin D and tear-film stability in dry eye disease: A case-control correlation study

**DOI:** 10.6026/973206300222360

**Published:** 2026-04-30

**Authors:** Anjali Singh, Pankaj Kushwaha, Pankaj Kumar Sharma

**Affiliations:** 1 Department of Ophthalmology, Chhindwara Institute of Medical Sciences, Chhindwara, Madhya Pradesh, India; 2 Department of Ophthalmology, Sadguru Sankalp Netra Chikitsalaya (SSNC) - Anandpur, Vidisha, Madhya Pradesh, India

**Keywords:** Dry eye disease (DED), vitamin D, tear-film break-up time (TFBUT), Schirmer test, ocular surface staining, tear meniscus height

## Abstract

Dry eye disease (DED) involves tear film instability, driving symptoms and epithelial damage, with emerging evidence linking vitamin D deficiency to ocular surface inflammation. Therefore, it is of interest to compare serum 25(OH) D levels and tear parameters in 300 DED eyes (150 patients) versus 270 control eyes (135 matched subjects) at a tertiary center (2022-2024). DED patients showed severe deficiency (mean 13.79±9.36 ng/mL vs 38.13±18.76 ng/mL in controls, p<0.0001), with OSDI 39.85±10.85, TFBUT 6.38±2.75s, ST-I 9.82±5.17 mm, TMH 0.19±1.21 mm, OSS 2.71±0.21; 25(OH)D correlated positively with TMH/ST-I/TFBUT (r=0.775/0.313/0.396) and negatively with OSDI (r=-0.414), all p<0.0001. Vitamin D deficiency is strongly associated with reduced tear volume and stability, as well as worse symptoms, particularly affecting TMH and epithelial integrity. Thus, we show vitamin D as a key modifier of the tear film, supporting its role as an adjuvant therapeutic target alongside standard management.

## Background:

Dry eye disease (DED) affects up to one-third of adults and is characterized by symptoms, tear-film instability and potential ocular surface damage [1, 2]. This synthesizes hyperosmolar stress, the homeostatic reflex and chronic inflammatory pathophysiology, the main part of which is immune activation at the innate and adaptable levels of the ocular surface [3, 4]. A growing body of literature places DED at the interface of cytokine cascades in T cells that trigger epithelial injury, goblet-cell depletion and neurosensory damage in mucosal tissues, thereby amplifying symptoms [3, 4- 5]. Vitamin D is also a secosteroid hormone with very extensive immunosuppressive actions beyond its effects on calcium metabolism. Through the vitamin D receptor, it influences epithelial differentiation, barrier integrity, innate antimicrobial peptide expression and tolerogenic programming of myeloid and lymphoid cells [6, 7]. Experimental work in ocular and periocular models shows that 1, 25-dihydroxyvitamin D can inhibit corneal Langerhans-cell migration, suppress proinflammatory cytokines and limit corneal neovascularization, consistent with a global dampening of ocular surface inflammation [6, 7- 8]. Exocrine epithelia, including salivary acini and other mucosal glands, are established vitamin D targets and fluid/ion transport is vitamin D-responsive suggesting plausible analogues in lacrimal gland physiology and tear-film regulation [6, 7-8]. Clinical evidence linking vitamin D status with DED, however, remains mixed. Multiple case control and cross-sectional studies report that individuals with DED have lower serum 25-hydroxyvitamin D levels than controls and that deficiency is associated with shorter tear-film breakup time (TFBUT), reduced Schirmer values and greater symptom burden [2, 4, 5, 6-7]. In contrast, some large population-based cohorts have found weaker or null associations after adjustment for age, sex, environmental exposures and comorbid autoimmune disease, raising the possibility that vitamin D may be a disease modifier rather than a primary causal factor [5, 6-7]. Interventional data albeit limited in sample size and duration suggest that cholecalciferol supplementation can improve TFBUT, Schirmer scores, tear osmolarity and patient-reported symptoms, particularly when added to standard topical therapy, yet effect sizes and target subgroups remain incompletely defined [6, 7-8]. It is also unclear whether vitamin D deficiency primarily influences aqueous tear production, evaporative instability, epithelial damage, or neurosensory components of the disease [9]. Against this background, we tested the hypothesis that serum 25-hydroxyvitamin D is associated in a graded fashion-with multiple tear-film domains (volume, stability, epithelial integrity) and with symptom burden in a case-control cohort of adults with and without DED [10, 11- 12]. Therefore, it is of interest to quantify which clinical parameters track most strongly with vitamin D status, with the dual aims of refining mechanistic interpretation and informing the design of future supplementation trials targeting clearly defined tear-film endophenotypes and clinically meaningful symptom outcomes.

## Materials and methods:

## Study design and setting:

This was a hospital-based case-control study conducted in the Department of Ophthalmology at Shyam Shah Medical College & Associated Gandhi Memorial Hospital, Rewa (Madhya Pradesh, India), from September 2022 to June 2024.

## Participants and sampling:

We enrolled 150 adults with dry eye disease (DED; 300 eyes) and 135 age- and sex-matched healthy controls (270 eyes) attending the tertiary center. Group sizes and matching are detailed in the thesis synopsis.

## Eligibility criteria:

Inclusion for cases required clinical DED based on symptoms and abnormal tear-film testing. Exclusions included contact-lens wear, ocular infection/allergy, prior ocular surgery, use of drugs altering tear production and systemic conditions listed in the data-collection proforma (e.g., connective-tissue disease, thyroid disease) to minimize confounding. Controls had no clinical evidence of DED.

## Clinical assessments:

All participants underwent a comprehensive ophthalmic evaluation, including best-corrected visual acuity, slit-lamp biomicroscopy, intraocular pressure and dilated fundus exam. DED severity was symptom-graded with the Ocular Surface Disease Index (OSDI; 0-100) using the standard scoring formula and cutpoints (normal 0-12; mild 13-22; moderate 23-32; severe >33). This synthesizes hyperosmolar stress, the homeostatic reflex and chronic inflammatory pathophysiology, the main part of which is immune activation at the innate and adaptable levels of the ocular surface [3, 4]. A growing body of literature places DED at the interface of cytokine cascades that trigger epithelial injury, goblet-cell depletion and neurosensory damage in mucosal tissues, thereby amplifying symptoms [3, 5]. Vitamin D is also a secosteroid hormone with very extensive immunosuppressive actions beyond its effects on calcium metabolism.

## Biochemistry:

Venous blood was fasted and serum 25-hydroxyvitamin D (25(OH) D) at the Central Processing Laboratory was measured using a chemiluminescence immunoassay (CLIA). The status of vitamin D was deficient (<20 ng/ mL), insufficient (20-30 ng/ mL) and sufficient (>30 ng/mL).

## Ethics and consent:

The aim of the study was clarified, confidentiality was ensured, informed consent was obtained from participants in writing and institutional permission was obtained.

## Statistical analysis:

The continuous variables are reported using mean ± SD and compared through t-tests. Categorical variables were summarized as counts (%) and compared using χ^2^ tests. Pearson correlation coefficients quantified associations between serum 25(OH) D and DED parameters. A two-sided p < 0.05 indicated statistical significance.

## Results:

Among cases, the mean OSDI was 39.85 ± 10.85; 48.67% had moderate symptoms (OSDI 23-32), 42.0% mild and 9.3% severe. Objective testing showed TMH <0.25 mm in ~70.7% of cases; mean TFBUT 6.38 ± 2.75s; Schirmer-1 9.82 ± 5.17 mm; and mean OSS 2.71 ± 0.21. Mean serum 25(OH) D was 13.79 ± 9.36 ng/mL in cases versus 38.13 ± 18.76 ng/mL in controls (p < 0.0001). Category distributions differed markedly: 67.33% of cases were deficient versus 11.11% of controls; sufficiency occurred in 11.33% of cases versus 71.11% of controls (overall χ^2^ p < 0.0001) (Table 1 - see PDF). In cases, higher serum 25(OH) D correlated with higher TMH (r = 0.775), TFBUT (r = 0.396), Schirmer-1 (r = 0.313) and OSS (r = 0.775) and inversely with OSDI (r = -0.414); all p < 0.0001. Mean 25(OH) D declined across OSDI strata (mild > moderate > severe) without statistical significance (p = 0.076). The ~24 ng/mL lower mean 25(OH) D in DED cases versus controls represents a large, clinically meaningful separation consistent with substantial systemic hypovitaminosis D burden among clinic DED patients. Table 2 (see PDF) shows a highly significant difference in vitamin D status between cases and controls (p < 0.0001). A large majority of cases (67.33%) were vitamin D deficient compared to only 11.11% of controls, indicating a strong association between deficiency and the condition under study. Conversely, most controls (71.11%) had sufficient vitamin D levels, while only 11.33% of cases were sufficient. The proportion of insufficient individuals was relatively comparable but still higher in cases. Overall, vitamin D deficiency appears markedly more prevalent among cases, suggesting it may be an important contributing or associated factor (Table 2 - see PDF). The percentage of categories shows how radical the change is to the left for DED patients (two- thirds deficient; approximately one-ninth sufficient) vs. controls (approximately 1 in 10 deficient; approximately 7 in 10 sufficient). The difference then indicates much higher risks of inadequacy in DED (relative odds 16) and much lower risks of adequacy. Table 3 (see PDF) demonstrates significant correlations between serum 25(OH)D levels and dry eye parameters (p < 0.0001 for all). OSDI shows a moderate negative correlation (r = -0.414), indicating that higher vitamin D levels are associated with fewer symptoms. TMH exhibits a strong positive correlation (r = 0.775), suggesting better tear volume with increasing vitamin D. Schirmer-1 and TFBUT show moderate positive correlations, reflecting improved tear production and stability. OSS also shows a strong positive correlation, indicating better ocular surface status with higher vitamin D levels. Overall, higher vitamin D levels are significantly associated with improved clinical and symptomatic dry eye parameters (Table 3 - see PDF). The table shows a decreasing trend in mean 25(OH)D levels with increasing OSDI severity, with the lowest levels observed in the severe group. This suggests that poorer vitamin D status may be associated with more severe dry eye symptoms. However, the difference is not statistically significant (p = 0.076), indicating that this trend should be interpreted cautiously and may not represent a definitive association (Table 4 - see PDF). Figure 1 (see PDF) describes the strong inclination towards left shift in the status of vitamin D in the cases of DED as compared to controls. Deficiency per cent (under 20 ng/mL) in two-thirds of patients in DED and adequate (above 20 ng/mL) in only approximately 11 per cent, compared to sufficient (above 20 ng/mL) in approximately 71 per cent of the controls the χ^2^ Test (p < 0.0001) demonstrates that the chance of such a distributional difference is highly unlikely through coincidence. At best, hypovitaminosis D, in this case, is clinically similar to DED when outcomes are extended to ocular- surface therapy as a routine supplement, as long as randomized evidence accumulates. Figure 2 (see PDF) depicts a large difference in serum 25(OH) D between DED cases (≈13.8 ng/mL) and the controls (≈38.1 ng/mL) with non-overlapping 95% confidence intervals and a highly significant t -test (p < 0.0001). The effect size reflected by the 24 ng/mL gap is large, consistent with the significantly greater burden of hypovitaminosis D among ED patients with DED. This has clinical support, as it advocates a regular vitamin D test in DED evaluation and indicates that it may be a reasonable supplement to ocular-surface treatments in cases of deficiency, pending randomized evidence.

## Discussion:

Our data demonstrate a marked difference in vitamin D status between DED cases and controls and show graded, statistically robust associations between serum 25(OH)D and multiple tear-film domains (volume, stability, epithelial staining) plus symptom burden. The strongest correlations were with TMH and epithelial staining, implicating fluid dynamics and epithelial barrier integrity as potential vitamin D-sensitive nodes. These findings align with several case-control and clinic-based studies that reported lower TFBUT and Schirmer scores, as well as worse symptoms, in individuals with vitamin D deficiency [13, 14, 15, 16, 17- 18]. Kurtul *et al.* reported significantly lower TFBUT and Schirmer scores in vitamin-D deficient participants compared with controls [13]. Yildirim *et al.* found positive correlations of 25(OH) D with Schirmer and TFBUT and inverse correlations with OSDI [14]. Demirci *et al.* also observed higher osmolarity and deficiency staining, which is characteristic of our relationship with OSS [15]. Jin *et al.* found positive associations between 25(OH) D and TFBUT, as well as Schirmer's test, in DED clinics [16]. Kaur *et al.* and Saha *et al.* reported convergent correlation of this in Indian cohorts [17, 18]. Compare with a population-based study by Jee *et al.* which reported a weak, insignificant independent association after multivariable correction [19]. The weakened result among population datasets might in part be connected with variations in case definition, or environmental exposures or residual confounding UV/sunlight, outdoor activity, adiposity, systemic disease), but the weakened outcome appears to be more because of clinic cohorts like those that we employ, such as sampling those who are at higher risk of DED and have stronger biological indicators. Experimentally, pathways are possible in that 1, 25-dihydroxyvitamin D limits dendritic-cell differentiation/migration and the synthesis of pro-inflammatory cytokines in corneal epithelia [8, 9-10]. Vitamin D-responsive is the exocrine transport of the epithelium, suggesting that the secretion in the lacrimal fluid can also be altered. Together with vitamin D's roles in epithelial junctions and antimicrobial peptides, this supports our finding that the vitamin D gradient is highest in TMH and OSS. Notably, small studies indicate that vitamin D supplementation can be used alongside traditional therapy. Bae *et al.* displayed evidence of TFBUT, tear production and symptom improvement in DED intractable to lubricants after intramuscular cholecalciferol [20]. Kizilgul *et al.* found a reduced tear osmolarity in deficiency during replacement [21]. Vitamin D status also affects the response to artificial tears and supplementation improves the situation [22]. We do not have an interventional study, but these interventional data could help strengthen biological plausibility and even support a conceivable clinical use. Weaknesses include that sampling is hospital-based, there is no meibography or tear osmolarity data for all subjects and confounding lifestyle factors (sun exposure, diet) are not modelled. It was not a stratified DED assignment based on an aqueous-deficient and evaporative phenotype; in this case, future studies should test whether one endotype, in particular, could have a preferential benefit in the vitamin D dose-response relationship.

## Conclusion:

DED patients exhibited profound vitamin D deficiency compared with controls, with higher 25(OH) D levels correlating with improved tear parameters (TMH/TFBUT/ST-I) and reduced OSDI/OS staining. Thus, vitamin D in epithelial barrier and tear secretion pathways is linked despite cross-sectional limitations. These data show vitamin D screening/repletion as an adjunct, pending RCTs to confirm causality, dosing and responders.
